# Glioblastoma multiforme with oligodendroglial component (GBMO): favorable outcome after post-operative radiotherapy and chemotherapy with nimustine (ACNU) and teniposide (VM26)

**DOI:** 10.1186/1471-2407-6-247

**Published:** 2006-10-18

**Authors:** Dirk Vordermark, Klemens Ruprecht, Peter Rieckmann, Wolfgang Roggendorf, Giles H Vince, Monika Warmuth-Metz, Oliver Kölbl, Michael Flentje

**Affiliations:** 1 Dept. of Radiation Oncology, University of Würzburg, Würzburg, Germany; 2 Dept. of Neurology, University of Würzburg, Würzburg, Germany; 3 Dept. of Neuropathology, University of Würzburg, Würzburg, Germany; 4 Dept. of Radiosurgery, University of Würzburg, Würzburg, Germany; 5 Dept. of Neuroradiology, University of Würzburg, Würzburg, Germany

## Abstract

**Background:**

The presence of an oligodendroglial component within a glioblastoma multiforme (GBM) is considered a prognostically favorable factor, but the clinical outcome of patients with glioblastoma multiforme with oligodendroglial component (GBMO) after combined post-operative radiotherapy and chemotherapy has rarely been reported.

**Methods:**

We analyzed overall and progression-free survival in a group of ten consecutive patients initially diagnosed with GBMO between 1996 and 2004 (4.2% of all GBM patients). Median (range) age was 54 (34–73) years, 90% were resected and median radiotherapy dose was 54 (45–60.6) Gy. 80% of patients received post-operative chemotherapy with nimustine (ACNU) and VM26 (teniposide) for a median of 3.5 (1–6) cycles, the remainder were treated with post-operative radiotherapy alone. All specimens were reviewed by an experienced neuropathologist.

**Results:**

Neuropathological re-evaluation revealed GBM with an oligodendroglial component of 30% or less in five cases, predominant oligoastrocytic tumors with focal areas of GBM in four patients and WHO grade III oligoastrocytoma with questionable transition to GBM in one patient. Four of ten patients were alive at at 40, 41, 41 and 82 months. The median overall survival (Kaplan-Meier) was 26 months, the 2-year survival rate was 60% (progression-free survival: 9.8 months and 40%, respectively).

**Conclusion:**

In conclusion, patients with GBMO treated with post-operative radiotherapy and chemotherapy with ACNU/VM26 had a better prognosis than reported for GBM in modern chemoradiation series.

## Background

Although glioblastoma multiforme with oligodendroglial component (GBMO) is not considered as a distinct disease entity in the most recent World Health Organization (WHO) classification of central nervous system tumors [1], the increased chemosensitivity and radiosensitivity of WHO grade III anaplastic oligodendrogliomas [2,3] has led to the assumption that patients with GBMO might similarly have a better prognosis than patients with classic glioblastoma multiforme (GBM). To the authors' knowldedge, data from phase III clinical trials on the outcome of the subgroup of patients with GBMO have not been published. In trial 83-02 of the Radiation Therapy Oncology Group (RTOG), a four-arm randomized phase I/II trial assessing different radiotherapy fractionation schedules with carmustine (BCNU) chemotherapy in all arms, the median survival of 14.3 months in GBMO was significantly longer than the 10.4 months observed in classic GBM [4]. The 2-year survival rates were 35% and 4%, respectively.

Until the recent establishment of combined chemoradiation with temozolomide as a new treatment standard [5], the use of chemotherapy in GBM patients was not generally recommended. A meta-analysis of randomized controlled trials of mostly nitrosurea-based chemotherapy in malignant glioma found an absolute increase in overall survival of 6% at one year and of 4% at two years in glioblastoma patients treated with chemotherapy [6]. In Germany, combination chemotherapy with nimustine (ACNU) and teniposide (VM26) was a preferred protocol in glioblastoma patients in whom chemotherapy was indicated, leading to a median overall survival of 17.3 months in glioblastoma in the NOA-01 trial [7]. It has been the policy in the authors' institution to recommend post-operative chemotherapy with ACNU and VM26, in addition to radiotherapy, if patients with GBMO were in sufficiently good general general condition (Karnofsky index of at least 70). We report here the clinical outcome of a series of ten consecutive patients with GBMO treated between 1996 and 2004.

## Methods

### Patients and treatment

The charts of all patients with a diagnosis of GBM referred to the Dept. of Radiation Oncology, University of Würzburg, were reviewed regarding a histopathological diagnosis of GBMO. For initial histopathological evaluation, immunohistochemistry for the markers glial fibirillary acidic protein (GFAP), Ki-67, p53 or CD68 was routinely performed by standard techniques. Although there is no uniform definition of GBMO, all patients in whom the initial histopathological report – on which treatment decisions were based – described a population of cells with oligodendroglial features next to a population of astrocytic and anaplastic glioma cells, with obvious features of highest malignancy typically found in GBM, were included in the study cohort, regardless of the extent of the oligodendroglial component [8]. Of 240 patients with GBM treated during the period studied, n = 10 (4.2%) were found to have an oligodendroglial component described in the initial pathology report. Histopathological slides of these ten patients were reviewed by an experienced neuropathologist (W. R.), who was blinded to the clinical outcome of the patients, on the basis of the WHO classification of 2000 [1]. The results of this re-evaluation did not affect inclusion of patients in the present analysis. The 1p/19q status was not determined as part of the present study.

A summary of patient and treatment characteristics for this group of GBMO patients is given in Table 1. Characteristics of individual patients are shown in Table 2. Patients were assigned to defined prognostic groups based on the RTOG recursive-partioning analysis data for malignant glioma [9]. Treatment details are listed in Tables 1 and 2. Radiotherapy was administered as a 3-D conformal treatment, the target volume being based on contrast-enhanced planning computed tomogrophy (CT) and pre-operative imaging. Chemotherapy with ACNU (90 mg/m^2 ^iv, day1) and VM26 (60 mg/m^2^, days 1 to 3; both drugs given in six-week intervals) was usually initiated after completion of radiotherapy. In general, it was aimed to administer six cycles of chemotherapy. In three patients, one or two cycles were given post-operatively before radiotherapy (Table 2).

### Statistics

Overall survival was studied as the main end point. Overall survival status was obtained from local resident's registration offices. Progression-free survival was used as a secondary endpoint. The dates of diagnostic imaging studies were considered in the calculation of progression-free survival. Progression was defined as an unequivocal increase in the size of the contrast-enhancing lesion on CT or magnetic resonance imaging (MRI), typically performed in three-month intervals, leading to a consideration of salvage treatment options in the interdisciplinary tumor board. Actuarial survival rates were calculated from the time of histological diagnosis using the Kaplan-Meier method. Patients consented to anonymous data analysis as part of the treatment contract with the university hospital.

## Results

**Figure 1:**
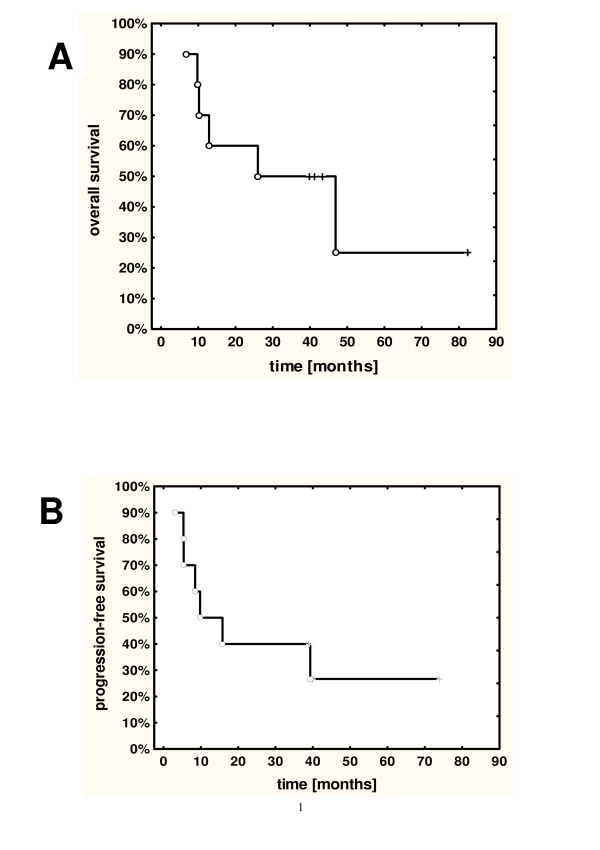
Overall survival (A) and progression-free survival (B) of n = 10 consecutive patients with glioblastoma multiforme with oligodendroglial component (GBMO).

The clinical course of individual patients is shown in Table 2. Eight of ten patients with GBMO received a median of 3.5 (range 1 to 6) cycles of chemotherapy with ACNU/VM26 as part of their adjuvant treatment. At the time of analysis, 4 of 10 patients were alive at 40, 41, 41 and 82 months. The Kaplan-Meier curves of overall survival and progression-free survival are shown in Fig. 1. The median overall survival of GBMO patients was 26 months. Overall survival at two years and at four years was 60% and 25%, respectively. The median progression-free survival was 9.8 months. At two and four years, 40% and 26.7% of patients, respectively, were free of progression.

Histopathological re-evaluation of all specimens by an experienced neuropathologist revealed different degrees of oligodendroglial component within the tumor tissue. The grading of results of immunohistochemistry for GFAP, Ki-67, p53 and CD68 in individual patients is given in Table 3. Whereas five tumors were classified as GBM with an oligodendroglial component of 30% or less, the others were diagnosed as containing a predominant WHO grade III oligodendroglial or oligoastrocytic component. In four of the latter group, features of WHO grade IV GBM were seen. In one case, for which only very limited biopsy material was available, anaplastic oligodendroglioma was diagnosed upon review without confirmation of GBM. The overall survival times for patients with GBM and oligodendroglial component of 30% or less were 10, 10, 13, 26 and 40+ months as compared to 7, 41+, 41+, 47, and 82+ months for patients with a predominant WHO grade III oligodendroglial or oligoastrocytic component.

## Discussion

Before the recent introduction of combined radiochemotherapy with temozolomide [5], the use of chemotherapy in GBM was not universally recommended. The presence of an oligodendroglial component within an astrocytic tumor with features of a WHO grade IV glioblastoma, however, has been regarded by some authors as an indicator of increased chemosensitivity of such tumors. This assumption was based on the high response rates of WHO grade III anaplastic oligodendroglioma, as compared to grade III anaplastic astrocytoma, established in phase II trials in the 1990s [2]. Two recent randomized phase III trials of adjuvant PCV in anaplastic oligodendroglioma and anaplastic oligoastrocytoma, RTOG 94-02 and EORTC 26951, found a significant improvement in progression-free survival (2.6 vs. 1.9 years and 2.0 vs. 1.1 years, respectively) after addition of PCV chemotherapy, but so far no effect on overall survival [10,11]. The clinical outcome of patients with WHO grade IV GBMO has rarely been reported. Median survival rates in the small series available in the literature (Table 4) ranged between 14 and 26 months, 2-year survival rates between 20% and 58%. The overall survival of 10 consecutive patients with GBMO in the present series (median 26 months, 60% at two years) compares favorably to these GBMO series and to the outcome of all GBM patients in large clinical trials, including the temozolomide radiochemotherapy arm in EORTC 26981 and the ACNU/VM26 arm in the NOA-01 trial [5,7].

The favorable survival rates in the present series of patients with GBMO may be a result of a good response of these tumors to initial multi-modality management with surgery, post-operative irradiation and chemotherapy with ACNU/VM26 (where feasible), but may also be influenced by the criteria for patient selection and the response to salvage therapy. Although the patients now analyzed were retrieved from the database of the radiation oncology department it appears unlikely that this introduced a relevant selection bias. Post-operative radiotherapy is a standard treatment in GBM and the reported median survival rate of all patients with GBM treated with radiotherapy in this department between 1990 and 1999 was 11.5 months [12]. The term GBMO has not been uniformly defined and especially the distinction of GBMO from anaplastic oligoastrocytoma is generally considered difficult [1,8,13]. However, nine of ten patients included clearly had tumors with components of highest malignancy, as found in grade IV GBM. In one patient with very limited biopsy material, the GBM component within a grade III oligoastrocytoma was judged as uncertain upon review. The fact that the patients now analyzed were actually considered to have GBM at the time of adjuvant treatment planning is underscored by the use of accelerated radiotherapy schedules, limiting the treatment duration to approximately three weeks, in 8 of 10 patients – an approach reserved for grade IV tumors at the authors' institution.

Within the limited patient cohort studied, the extent of the oligodendroglial component appeared to be linked to prognosis. Whereas only one of five patients with 30% or less oligodendroglial cells survived for more than two years, this was true for four of five patients with a predominant oligodendroglial component. Obviously, such quantifications must be regarded with caution as percentages for particular cell types on sectioned material need not be representative of the overall tumors.

GBMO tumors have been reported to frequently contain molecular genetic alterations associated with increased chemosensitivity in oligodendroglial tumors such as a loss of heterozygosity (LOH) on chromosome arms 1p and 19q [8,13]. Microarray analysis of gene expression has been suggested as a method to distinguish "classic GBM" tumors from a group termed "non-classic" GBM with a better prognosis [14]. In the present series, identification of an oligodendroglial component on routine histopathological evaluation of tumor sections was apparently able to select a patient group with a better prognosis than in classic GBM.

In some of the previous series of patients with GBMO, patients were mostly treated with surgery and post-operative radiotherapy, but without chemotherapy. This may in part explain the favorable results of the present analysis. The low rate of patients surgically treated with biopsy only (1/10 in the present series) is comparable to the series of Kraus et al. [8] (Table 3) where all patients were resected such that a particularly aggressive surgical approach in the present patient group appears to be an unlikely explanation of the high survival rates.

The progression-free survival observed may be regarded as a better indicator of the efficacy of the initial treatment regimen. The median progression-free survival of 9.8 months is also longer than that seen in large trials of GBM, e. g. 6.9 months in the temozolomide arm of EORTC 26981 [5]. This suggests that the response of GBMO to ACNU/VM26 might be better than that of GBM in general to temozolomide. Clearly, the use of salvage treatments including surgery, stereotactic hypofractionated reirradiation [15] and temozolomide chemotherapy has led to prolonged survival in individual patients failing early after initial post-operative chemotherapy with ACNU/VM26 (Table 2). The efficacy of temozolomide as an initial treatment or as salvage therapy for GBMO has not been published to our knowledge.

The information gained by the present study is limited by the small number of patients who, nevertheless, represent the total number of this entitity from an eight-year period at a university center. A similar analysis focussing on specific effects of the addition of chemotherapy in patients with GBMO would be possible in subsets of patients from recently completed randomized trials in glioblastoma. It would, therefore, be of interest to analyze specifically the clinical course of GBM patients with an oligodendroglial component treated with temozolomide in EORTC 26981. It is also unknown whether patients with GBMO might benefit from an escalation of radiation dose, an approach currently considered not benefitial in GBM [16], or from high-dose chemotherapy protocols as investigated in anaplastic oligodendroglioma [17].

## Conclusion

In summary, the prognosis of patients with GBMO treated with post-operative radiotherapy and chemotherapy with ACNU/VM26 was favorable compared to published data for GBMO treated mostly with post-operative radiotherapy alone and compared to GBM treated with post-operative radiotherapy and different regimens of chemotherapy. The outcome of GBMO after radiotherapy with concomitant and adjuvant temozolomide, the current standard for GBM, is not known. A superiority of ACNU/VM26 over temozolomide in GBMO can not be advocated. From our experience, post-operative chemotherapy should be offered to patients with GBM whenever possible in the presence of an oligodendroglial component.

## Competing interests

The author(s) declare that they have no competing interests.

## Authors' contributions

DV designed the analysis, reviewed patient data, performed statistical analysis and drafted the manuscript.

KR treated the patients, reviewed patient data and revised the manuscript.

PR treated the patients, reviewed patient data and revised the manuscript.

WR performed neuropathological evaluation and revised the manuscript.

GHV treated the patients, reviewed patient data and revised the manuscript.

MWM performed imaging studies on the patients, reviewed patient data and revised the manuscript.

OK treated the patients, reviewed patient data and revised the manuscript.

MF treated the patients, reviewed patient data and revised the manuscript.

All authors read and approved the final manuscript.

## Pre-publication history

The pre-publication history for this paper can be accessed here:



## Figures and Tables

**Table 1:** Overview of patient and treatment characteristics

age at diagnosis (years)	*median (range)*	54 (34–73)
surgical procedure	*biopsy resection*	1/10 (10%) 9/10 (90%)
total RT dose (Gy)	*median (range)*	54 (45–60.6)
adjuvant chemotherapy with ACNU/VM26		8/10 (80%)
no. of adjuvant cycles	*median (range)*	3.5 (1–6)
≥ 4 adjuvant cycles		4/10 (40%)

Overview of patient and treatment characteristics for n = 10 consecutive patients with glioblastoma multiforme with oligodendroglial component (GBMO).

**Table 2:** Characteristics, treatment and clinical course of individual patients

**RPA class**	**no.**	**sex**	**age (years)**	**result of neuropathologic reevaluation (% of oligodendroglial component, where applicable)**	**surgery**	**total/daily RT dose (Gy)**	**total/pre-RT adjuv. cycles ACNU/VM26 (reason for termination)**	**salvage treatment**	**overall survival (months)**
III	1	m	34	GBM with extensive oligodendroglial component (30%)	resection	55.1/2 × 1.8	6	--	40+
	2	f	39	grade III oligoastrocytoma with transition to GBM	resection	60.6/2 × 1.8	3 (thrombo-cytopenia III°)	17 × TEM	41+
IV	3	f	51	small-cell GBM with extensive oligodendroglial component (75%)	resection	53.4/2 × 1.8	2/1 (leuko-cytopenia II°)	1 × ACNU/VM26	47
	4	m	53	anaplastic oligodendroglioma with focal transition to GBM	resection	59.4/1.8	5/1	--	82+
	5	f	73	GBM with oligodendroglial component (30%)	resection	59.4/1.8	4/2	reirradiation (6 × 5 Gy), surgery, 1 × TEM	26
V	6	f	54	anaplastic oligodendroglioma with transition to GBM	resection	45/3	6	--	41+
	7	f	62	GBM with minor oligodendroglial component	resection	54/2 × 1.8	2 (progression)	3 × TEM	13
	8	f	54	GBM with oligodendroglial component (20%)	resection	45/3	1 (thrombo-cytopenia IV°)	--	10
VI	9	m	60	very small tissue sample, anaplastic oligodendroglioma, GBM possible	open biopsy	54/2 × 1.8	--	--	7
	10	m	72	GBM with minor oligodendroglial component (less than 5%)	resection	45/3	--	--	10

Patient and treatment characteristics and clinical course of n = 10 consecutive patients with glioblastoma multiforme with oligodendroglial component. The results of the neuropathological re-evaluation at the time of the present analysis are summarized (GBMO; RPA: recursive partitioning analysis according to [9], RT: radiotherapy, ACNU: nimustine, VM26: teniposide, TEM: temozolomide).

**Table 3:** Results of immunohistochemistry in individual patients with GBMO

**patient no.**	**GFAP**	**Ki-67**	**p53**	**CD68**
1	++	+	++	n. a.
2	+	+++	+	++
3	++	++	n. a.	+
4	n. a.	n. a.	n. a.	n. a.
5	++	+	n. a.	++
6	+	+++	n. a.	n. a.
7	+	++	+	++
8	+	++	+	++
9	++	+	n. a.	++
10	+	++	n. a.	n. a.

Results of immunohistochemistry for glial fibrillary acidic protein (GFAP), Ki-67, p53 and CD68 in individual patients with glioblastoma multiforme with oligodendroglial component (GBMO; patient numbers correspond to Table 2). Staining was scored as weak (+), intermediate (++), strong (+++) or not available (n.a.).

**Table 4:** Treatment results of selected series of GBM and GBMO

**series**	**patient group**	**n**	**treatment**	**median overall survival (months)**	**2-year overall survival**	**reference**
NOA-01	all GBM	154	post-OP RT + ACNU/VM26	17.3	25%	[7]
EORTC 26981	all GBM	287	post-OP RT + TEM	14.6	26.5%	[5]
RTOG 83-02	GBMO	23	post-OP RT + BCNU	14.3	(3-year survival: 35%)	[4]
retrospective	GBMO	12	resection + RT, chemo in 2/12	26	58% (data taken from graph)	[8]
retrospective	GBMO	11	post-OP RT in 65% (total group)	17.7	20% (data taken from graph)	[18]
retrospective	GBMO	5	post-OP RT in 80%	16.2	n. a.	[19]
**present series**	**GBMO**	**10**	**post-OP RT + ACNU/VM26 (8/10)**	**26**	**60%**	

Overview of treatment results in glioblastoma multiforme (GBM) and GBM with oligodendroglial component (GBMO) from selected series (RT: radiotherapy, ACNU: nimustine, VM26: teniposide, TEM: temozolomide, n. a.: not available).
